# When Common Becomes Normal: Weaker Association Between Neighborhood Stress and Body Mass Index Among Black Adolescents Compared to White Adolescents

**DOI:** 10.31586/gjcd.2024.1121

**Published:** 2024-11-14

**Authors:** Shervin Assari, Hossein Zare

**Affiliations:** 1 Department of Internal Medicine, Charles R. Drew University of Medicine and Science, Los Angeles, CA, United States; 2 Department of Family Medicine, Charles R. Drew University of Medicine and Science, Los Angeles, CA, United States; 3 Department of Urban Public Health, Charles R. Drew University of Medicine and Science, Los Angeles, CA, United States; 4 Marginalization-Related Diminished Returns (MDRs) Center, Los Angeles, CA, United States; 5 Department of Health Policy and Management, Johns Hopkins Bloomberg School of Public Health, Baltimore, MD, United States; 6 School of Business, University of Maryland Global Campus (UMGC), Adelphi, MD, United States

**Keywords:** Neighborhood Stress, Body Mass Index (BMI), Perceived Safety, Fear, Adolescents, Racial Disparities, Black Youth, White Youth, Obesity, Environmental Stressors

## Abstract

**Objective:**

This study explores the relationship between neighborhood stress and Body Mass Index (BMI) in adolescents, while also examining whether this association differs between Black and White adolescents.

**Methods:**

Data from the Adolescent Brain Cognitive Development (ABCD) Study were analyzed using linear regression models to examine the association between neighborhood stress (defined as a composite score based on three items measuring perceived safety and neighborhood fear) and BMI in adolescents, controlling for demographic and socioeconomic variables. We tested models both with and without interaction terms to assess whether race moderated the association. Stratified analyses were conducted to further explore potential differences between Black and White adolescents.

**Results:**

A positive association was observed between neighborhood stress and BMI across the overall sample. However, this association was weaker for Black adolescents compared to White adolescents, even after adjusting for potential confounders.

**Conclusions:**

The contribution of neighborhood stress to higher BMI in adolescents may vary by race. Our findings suggest that while neighborhood stress is associated with increased BMI, Black adolescents appear to be less affected by these stressors than their White peers. This weaker association could be due to the normalization of stress in environments where it is pervasive (what is common becomes normal) or the presence of other significant risk factors affecting BMI in Black youth, such as poverty, limited food access, food culture, and food deserts. Future research should explore processes of habituation, inoculation, or even sensitization to stress among Black populations, who are often exposed to a wide range of stressors throughout the life course.

## 1. Introduction

The rising prevalence of obesity and overweight among adolescents has become a significant public health concern [1]. Body Mass Index (BMI), a primary indicator of obesity, is influenced by various environmental and psychosocial factors, including neighborhood conditions [2]. Adolescents living in neighborhoods characterized by high levels of stress—such as low perceived safety and heightened fear—are often at greater risk for higher BMI [3,4]. Stressful environments can encourage unhealthy behaviors, including poor dietary choices, reduced physical activity, and disrupted sleep patterns, all of which contribute to weight gain [5,6].

Previous research suggests that the impact of neighborhood stress on health outcomes, including BMI, may vary across racial and ethnic groups [7]. Black youth, in particular, are more likely to live in neighborhoods with higher levels of stress compared to their White peers [8]. Despite this, Black adolescents may not exhibit greater sensitivity to these adversities in terms of health outcomes [9]. Some studies have found that stress has weaker health effects on Black adolescents than on White adolescents, suggesting that race may moderate the impact of environmental stressors on BMI [9].

Social groups facing higher adversity may exhibit reduced sensitivity to new stressors. This phenomenon, known as “inoculation” [10–12] or “stress habituation” [13], suggests that repeated exposure to challenges can dampen typical stress responses, potentially mitigating effects on outcomes like weight gain [14–16]. For Black adolescents, frequent exposure to stressors, including economic hardship, structural racism, and environmental disadvantages, may foster a form of resilience or adaptive coping [17]. Consequently, the physiological and psychological effects of neighborhood stress, such as elevated BMI, may be less pronounced compared to their White peers—a phenomenon sometimes described as “what is common may become normal.” Additionally, resilience derived from factors like strong family support systems, cultural identity, or community bonds may further reduce the impact of stressors for Black individuals [19–22]. These protective elements can buffer against the negative effects of high-stress environments, thus diminishing the influence of neighborhood stress on adverse health outcomes like obesity. Analyzing the impact of environmental stressors on Black populations’ health requires attention to both structural inequities and the resilience that helps navigate these significant challenges [23–30].

Drawing on data from the Adolescent Brain Cognitive Development (ABCD) Study [31–36], the first aim of the present study seeks to explore the relationship between neighborhood stress and BMI in adolescents. Our second aim is to investigate whether this association differs by race. We hypothesize that neighborhood stress will be positively associated with BMI for all adolescents in the pooled sample. However, we anticipate that the strength of this association between neighborhood stress and BMI will be weaker for Black adolescents compared to White adolescents. This may be due to habituation, as well as the more substantial influence of other structural factors on BMI in Black adolescents. Given the pervasive exposure to stressors in the lives of Black adolescents, each additional unit of exposure to a specific type of stress may exert a weaker effect on BMI within this group. Due to high levels of stress across a range of sources, Black adolescents may have developed and mobilized adaptive or resilience mechanisms in response to chronic stress exposure.

## 2. Methods

### 2.1. Study Population

This study utilized data from the Adolescent Brain Cognitive Development (ABCD) Study [31–36], a large-scale longitudinal study that tracks cognitive and health-related outcomes among adolescents across the United States. The ABCD dataset includes a racially and socioeconomically diverse sample, providing a rich resource for examining the effects of environmental factors on youth development. For the purposes of this analysis, the sample was restricted to adolescents who self-identified as either Black or White and who had complete data available for Body Mass Index (BMI), neighborhood stress measures, and relevant covariates. Adolescents from other racial or ethnic backgrounds were excluded to allow for a focused comparison between Black and White youth.

### 2.2. Measures

Body Mass Index (BMI), the primary outcome variable, was derived using self-reported height and weight data, which were converted into BMI (kg/m^2^) following standard procedures. BMI was treated as a continuous variable in the analyses, allowing for a nuanced examination of how neighborhood stress (score) correlates with adolescent BMI score.

Neighborhood stress, the key independent variable, was operationalized through measures of perceived safety and fear within the neighborhood. Perceived safety was assessed using three questions: 1) “I feel safe walking in my neighborhood, day or night,” 2) “Violence is not a problem in my neighborhood,” and 3) “My neighborhood is safe from crime.” These items captured parents’ feelings of safety while walking in the neighborhood and their concerns about crime and violence. The three items were combined into a composite index to create a robust measure of neighborhood stress, with higher values indicating greater perceived neighborhood stress (i.e., lower perceived neighborhood safety). To enhance the validity and reliability of this measure, parents (who reported on neighborhood safety) were instructed to consider their neighborhood as the area within about a 20-minute walk (or approximately one mile) from their home. For each statement, participants chose from the following responses: 1) strongly agree, 2) agree, 3) neither agree nor disagree, 4) disagree, or 5) strongly disagree.

Race/ethnicity was self-reported by the adolescents and categorized as either Black or White. This racial categorization allowed for the examination of potential differences in how neighborhood stress affects BMI across these two groups.

Covariates included neighborhood income (divided by 5000 to yield a more interpretable coefficient), gender, age, physical activity, family structure, and family socioeconomic status.

### 2.3. Statistical Analysis

The relationship between neighborhood stress and BMI was analyzed using linear regression models. These models assessed the overall effect of the composite neighborhood stress index on BMI, while controlling for potential confounding factors. Covariates included age, gender, family structure, and socio-economic status (measured via parental education and household income). These variables were included in the models to control for their potential correlation with both neighborhood stress and BMI. To explore whether the association between neighborhood stress and BMI varied by race, an interaction term was included in the regression models to formally test whether race moderated the relationship between neighborhood stress and BMI. This approach allowed for a clear comparison of the strength and direction of the association across racial groups. Linear regression models were fitted in a stepwise manner. In the first step, unadjusted models were estimated to examine the direct bivariate relationship between neighborhood stress and BMI. Next, models included socio-economic variables (parental education, household income) and demographic factors to account for potential confounding by demographic and SES factors. All analyses were conducted using the statistical software Stata. Model assumptions, including normality and homoscedasticity of residuals, were checked and addressed where necessary. Statistical significance was determined at the p < 0.05 level, and effect sizes were reported alongside p-values to quantify the strength of associations.

### 2.4. Ethical Considerations

The ABCD Study [31–36] received approval from institutional review boards (IRBs) at participating institutions, ensuring that data collection adhered to strict ethical standards. All data used in this study were de-identified, ensuring the privacy and confidentiality of the participants. Informed consent was obtained from the adolescents’ parents or legal guardians, while assent was obtained from the adolescents themselves prior to their participation in the study. The use of the ABCD data for this secondary analysis was approved by the relevant data use agreements, and ethical guidelines for secondary data analysis were followed throughout the research process.

## 3. Results

Overall, 10,764 youth entered our analysis who were either White (n = 7,723) or Black (n = 2,164). In the overall sample, 76.7% (SE = 0.004, 95% CI: 75.8%, 77.4%) of the adolescents were White, and 23.3% (SE = 0.004, 95% CI: 22.6%, 24.2%) were Black. Among the overall sample, 47.7% (SE = 0.005, 95% CI: 46.8%, 48.7%) of the participants were female, while 52.3% (SE = 0.005, 95% CI: 51.3%, 53.2%) were male. For White adolescents, 47.1% (SE = 0.005, 95% CI: 46.1%, 48.2%) were female, and 52.9% (SE = 0.005, 95% CI: 51.8%, 53.9%) were male. Among Black adolescents, 49.6% (SE = 0.010, 95% CI: 47.7%, 51.6%) were female, while 50.4% (SE = 0.010, 95% CI: 48.4%, 52.3%) were male. For the overall sample, 32.0% (SE = 0.004, 95% CI: 31.1%, 32.9%) of the adolescents were from unwed households, while 68.0% (SE = 0.004, 95% CI: 67.1%, 68.9%) were from married households. Among White adolescents, 21.5% (SE = 0.005, 95% CI: 20.6%, 22.4%) were from unwed households, and 78.5% (SE = 0.005, 95% CI: 77.6%, 79.4%) were from married households. For Black adolescents, 66.5% (SE = 0.009, 95% CI: 64.6%, 68.3%) were from unwed households, and 33.5% (SE = 0.009, 95% CI: 31.7%, 35.4%) were from married households.

Table 1 presents the descriptive statistics for the overall sample as well as for White and Black adolescents, focusing on key variables such as neighborhood stress, age, total family income, physical activity, neighborhood income, and Body Mass Index (BMI).

For the overall sample, adolescents reported an average neighborhood stress score of 2.06 (SE = 0.01, 95% CI: 2.04, 2.08). The mean BMI was 18.70 (SE = 0.04, 95% CI: 18.62, 18.78), while the average total family income was 7.32 (SE = 0.02, 95% CI: 7.27, 7.36). Physical activity had an average score of 3.57 (SE = 0.02, 95% CI: 3.52, 3.61), and the mean neighborhood income (measured in increments of $50,000) was 0.73 (SE = 0.00, 95% CI: 0.72, 0.74). The average age of participants was 9.48 years (SE = 0.01, 95% CI: 9.47, 9.49).

For White adolescents, neighborhood stress was lower, with a mean score of 1.92 (SE = 0.01, 95% CI: 1.90, 1.93), and the mean BMI was 18.22 (SE = 0.04, 95% CI: 18.14, 18.31), which is slightly below the overall sample. Total family income was higher for White adolescents, with an average of 7.86 (SE = 0.02, 95% CI: 7.82, 7.91). Physical activity levels were slightly higher among White adolescents, with a mean score of 3.68 (SE = 0.03, 95% CI: 3.63, 3.73), and the mean neighborhood income was also higher at 0.82 (SE = 0.00, 95% CI: 0.81, 0.83). The average age for White adolescents was nearly identical to the overall sample at 9.48 years (SE = 0.01, 95% CI: 9.47, 9.49).

For Black adolescents, the average neighborhood stress was significantly higher, with a mean of 2.58 (SE = 0.02, 95% CI: 2.54, 2.63). Black adolescents also had a higher mean BMI of 20.39 (SE = 0.11, 95% CI: 20.17, 20.62), which was notably above the overall and White means. Total family income for Black adolescents was considerably lower, with an average of 5.36 (SE = 0.06, 95% CI: 5.25, 5.47). Physical activity scores were lower among Black adolescents, with a mean of 3.16 (SE = 0.05, 95% CI: 3.06, 3.26). Additionally, the average neighborhood income was significantly lower for Black adolescents at 0.43 (SE = 0.01, 95% CI: 0.41, 0.45). The average age of Black adolescents was 9.47 years (SE = 0.01, 95% CI: 9.45, 9.50), similar to the overall sample and White adolescents.

The correlations between key study variables and Body Mass Index (BMI) for the entire sample, as well as stratified by race (Black and White adolescents), are presented in Table 2. For the overall sample, neighborhood stress was positively correlated with BMI (r = 0.14, *p* < 0.05), indicating that higher levels of perceived neighborhood stress were associated with higher BMI among adolescents. Additionally, being Black was significantly correlated with higher BMI (r = 0.21, *p* < 0.05), while higher family income (r = −0.26, *p* < 0.05), living in a married household (r = −0.20, *p* < 0.05), and greater neighborhood income (r = −0.18, *p* < 0.05) were all negatively correlated with BMI, suggesting that these factors are associated with lower BMI. There were no significant correlations between BMI and physical activity or age in the overall sample.

For White adolescents, neighborhood stress was also positively correlated with BMI (r = 0.11, *p* < 0.05), although the strength of this relationship was weaker compared to the overall sample. Other significant correlations included a negative association between family income and BMI (r = −0.22, *p* < 0.05) and between living in a married household and BMI (r = −0.16, *p* < 0.05). Additionally, neighborhood income was negatively correlated with BMI (r = −0.13, *p* < 0.05). No significant correlations were found between BMI and physical activity or age in White adolescents.

In the Black adolescent sample, the relationship between neighborhood stress and BMI was much weaker (r = 0.05, *p* > 0.05), and not statistically significant. Notably, the only variable significantly correlated with BMI in this group was age (r = 0.07, *p* < 0.05), indicating a small but positive relationship between older age and higher BMI. Unlike the White sample, family income, household composition, and neighborhood income were not significantly correlated with BMI in Black adolescents.

These results suggest that while neighborhood stress is generally associated with higher BMI in adolescents, the strength of this association may differ across racial groups, with the correlation being weaker and non-significant among Black adolescents. Additionally, socioeconomic factors, such as family income and neighborhood characteristics, appear to play a larger role in influencing BMI among White adolescents compared to Black adolescents.

The results from the linear regression models examining the relationship between neighborhood stress and Body Mass Index (BMI) in adolescents are presented in Table 3. In Model 1, neighborhood stress was positively and significantly associated with BMI. Specifically, for every unit increase in neighborhood stress, BMI increased by 0.620 units (SE = 0.040, 95% CI [0.542, 0.697], *p* < 0.001), indicating that adolescents living in more stressful environments tend to have higher BMI.

In Model 2, we included demographic and socioeconomic control variables. The positive association between neighborhood stress and BMI weakened substantially and became non-significant (B = 0.078, SE = 0.047, 95% CI [−0.015, 0.170], *p* = 0.099) when adjusting for factors such as age, gender, family income, marital status of parents, physical activity, and neighborhood income. Among the control variables, older age was significantly associated with higher BMI (B = 0.670, SE = 0.079, 95% CI [0.515, 0.826], *p* < 0.001). Being male was associated with a slightly lower BMI (B = −0.181, SE = 0.080, 95% CI [−0.338, −0.024], *p* = 0.024). Total family income and living in a married household were both negatively associated with BMI, with higher family income and marital status associated with lower BMI (B = −0.272, SE = 0.023, 95% CI [−0.316, −0.227], *p* < 0.001 for family income; B = −0.488, SE = 0.108, 95% CI [−0.698, −0.277], *p* < 0.001 for married household). Additionally, higher levels of physical activity were associated with lower BMI (B = −0.042, SE = 0.018, 95% CI [−0.076, −0.007], *p* = 0.017), as was higher neighborhood income (B = −0.472, SE = 0.107, 95% CI [−0.681, −0.263], *p* < 0.001). Being Black was associated with significantly higher BMI compared to White adolescents (B = 1.010, SE = 0.113, 95% CI [0.789, 1.232], *p* < 0.001).

In Model 3, we examined the interaction between race and neighborhood stress. The interaction term between being Black and neighborhood stress was significant (B = −0.207, SE = 0.094, 95% CI [−0.390, −0.023], *p* = 0.027), indicating that the effect of neighborhood stress on BMI was weaker for Black adolescents compared to White adolescents. In this model, neighborhood stress regained statistical significance, with an effect size of B = 0.145 (SE = 0.056, 95% CI [0.035, 0.255], *p* = 0.010), suggesting that neighborhood stress continues to have a positive association with BMI, though the strength of this association varies by race. For Black adolescents, the overall relationship between neighborhood stress and BMI was significantly weaker than for White adolescents.

## 4. Discussion

The results of this study provide suggestive evidence that neighborhood stress, composed of perceived lack of safety and fear, is positively associated with higher BMI in adolescents. However, this relationship was notably weaker for Black adolescents compared to White adolescents.

Our first finding—linking fear of crime and elevated BMI—is consistent with a well-established body of literature showing that environmental stressors, such as crime-related fear, are associated with obesity [37–39]. Numerous studies have documented higher BMI among adults, children, adolescents, and older adults residing in disadvantaged or unsafe neighborhoods. One plausible explanation is that individuals in unsafe neighborhoods may limit their outdoor activities, such as walking, jogging, or engaging in other forms of exercise, particularly after dark. Concerns about personal safety may lead to a reduction in physical activity, a known risk factor for increased BMI. Moreover, an unsafe neighborhood may serve as a proxy for other environmental deficits, such as a lack of green spaces, poor walkability, and fewer recreational facilities, all of which contribute to sedentary behavior and weight gain [40–43].

Our second finding, that the association between neighborhood stress and BMI is weaker for Black adolescents, aligns with previous research suggesting that Black youth, despite often being exposed to higher levels of environmental stress, do not always exhibit stronger associations between these stressors and negative health outcomes, such as obesity, compared to their White counterparts [9,44].

Several explanations may account for the observed weaker effect in Black adolescents compared to their White peers. One possibility is that Black adolescents develop adaptive coping strategies over time in response to chronic exposure to environmental stressors [45]. This prolonged exposure to various stressors may foster psychological resilience, potentially reducing the impact of stress on health outcomes like BMI [46–48]. When exposure to unsafe or disordered neighborhoods becomes a regular part of life, individuals may become desensitized to negative stimuli, which can lessen its physiological and psychological effects [49–51]. This phenomenon, known as “inoculation” or “stress habituation,” suggests that repeated exposure to adversity may blunt typical stress responses, thereby mitigating its impact on weight gain.

Another explanation for this racial disparity lies in the socio-cultural context [52]. Resilience within Black communities, as noted in prior research, may stem from frequent exposure to a range of challenges, including economic hardships, discrimination, and neighborhood disadvantage [53–58]. Coping mechanisms, whether collective or individual, such as strong family ties, community support, or spiritual practices, could buffer the impact of environmental stressors [52,59–61]. This adaptive resilience may contribute to Black adolescents being less vulnerable to stress-induced weight gain than their White peers, who may not face the same degree of chronic adversity [9,62].

Structural racism and socio-economic inequalities play a significant role in shaping both neighborhood conditions and health outcomes [63,64]. Black adolescents are disproportionately represented in economically disadvantaged neighborhoods, which often lack resources such as healthy food options, recreational facilities, and accessible healthcare services. These structural inequities may complicate the relationship between neighborhood stress and BMI by influencing lifestyle behaviors, such as diet and physical activity, that contribute to weight gain [39,65–67]. Additionally, persistent stress due to structural barriers may drive unhealthy coping behaviors, such as emotional eating, which may have differential impacts across racial groups [9,62].

These findings highlight the need for a more nuanced understanding of how neighborhood stress influences health across racial and ethnic groups. While stress is an important determinant of health, the factors that mediate or moderate its effects likely differ by race. Black adolescents, for instance, may be exposed to both protective and risk factors that interact with environmental stressors in complex ways. Identifying the specific protective factors—whether social, cultural, or psychological—that buffer the effects of neighborhood stress on BMI is an important avenue for future research. Doing so could inform targeted interventions aimed at addressing obesity and promoting health equity among adolescents of all racial and ethnic backgrounds.

In conclusion, while high perceived neighborhood stress is significantly associated with higher BMI in adolescents overall, this effect is not uniform across racial groups. Black adolescents experience a weaker association between neighborhood stress and BMI compared to their White peers, possibly due to the pervasive presence of various forms of stress in their lives, which may reduce the impact of each individual stressor. This aligns with the idea that what is common may become normalized. Addressing the broader structural and environmental factors contributing to health disparities is essential for developing effective public health interventions. Targeted strategies that both promote resilience and reduce neighborhood stress impacts are crucial for addressing obesity and improving well-being among all adolescents.

## Figures and Tables

**Table 1.** Descriptive Data Overall and by Race

	All				White				Black			
	Mean	SE	95% CI		Mean	SE	95% CI		Mean	SE	95% CI	
Baseline Neighborhood Stress*	2.06	0.01	2.04	2.08	1.92	0.01	1.90	1.93	2.58	0.02	2.54	2.63
Baseline Age (Year)	9.0	0.01	9.0	9.0	9.0	0.01	9.0	9.0	9.0	0.01	9.0	9.0
Baseline Total Family Income* (1–10)	7.32	0.02	7.27	7.36	7.86	0.02	7.82	7.91	5.36	0.06	5.25	5.47
Physical Activity*	3.57	0.02	3.52	3.61	3.68	0.03	3.63	3.73	3.16	0.05	3.06	3.26
Baseline Neighborhood Income / 5000*	0.73	0.00	0.72	0.74	0.82	0.00	0.81	0.83	0.43	0.01	0.41	0.45
Baseline Body Mass Index (BMI) *	18.70	0.04	18.62	18.78	18.22	0.04	18.14	18.31	20.39	0.11	20.17	20.62
	%	SE	95% CI		%	SE	95% CI		%	SE	95% CI	
Race												
White	0.767	0.004	0.758	0.774								
Black	0.233	0.004	0.226	0.242								
Sex												
Female	0.477	0.005	0.468	0.487	0.471	0.005	0.461	0.482	0.496	0.010	0.477	0.516
Male	0.523	0.005	0.513	0.532	0.529	0.005	0.518	0.539	0.504	0.010	0.484	0.523
Baseline Household Structure*												
Not Married	0.320	0.004	0.311	0.329	0.215	0.005	0.206	0.224	0.665	0.009	0.646	0.683
Married	0.680	0.004	0.671	0.689	0.785	0.005	0.776	0.794	0.335	0.009	0.317	0.354

* p < 0.05

**Table 2.** Correlations Overall and by Race (Pearson)

	1	2	3	4	5	6	7	8	9
**All**									
1 Black	1.00								
2 Neighborhood Stress	0.30*	1.00							
3 Male	−0.02	−0.02	1.00						
4 Age	−0.01	−0.03*	0.02	1.00					
5 Total Family Income	−0.44*	−0.37*	0.00	0.04*	1.00				
6 Married Household	−0.41*	−0.25*	0.01	0.02	0.56*	1.00			
7 Physical Activity	−0.10*	−0.07*	0.05*	0.07*	0.14*	0.10*	1.00		
8 Neighborhood Income / 50000	−0.37*	−0.39*	0.01	0.04*	0.45*	0.30*	0.11*	1.00	
9 Body Mass Index (BMI)	0.21*	0.14*	−0.03*	0.07*	−0.26*	−0.20*	−0.06*	−0.18*	1.00
**White**									
1 Black	-	-							
2 Neighborhood Stress	-	1.00							
3 Male	-	−0.04*	1.00						
4 Age	-	−0.03*	0.02	1.00					
5 Total Family Income	-	−0.27*	−0.01	0.04*	1.00				
6 Married Household	-	−0.13*	0.00	0.01	0.45*	1.00			
7 Physical Activity	-	−0.05*	0.05*	0.08*	0.12*	0.06*	1.00		
8 Neighborhood Income / 50000	-	−0.28*	0.01	0.03*	0.33*	0.15*	0.07*	1.00	
9 Body Mass Index (BMI)	-	0.11*	0.00	0.07*	−0.22*	−0.16*	−0.06*	−0.13*	1.00
**Black**									
1 Black	-								
2 Neighborhood Stress	-	1.00							
3 Male	-	0.04*	1.00						
4 Age	-	−0.03*	0.03*	1.00					
5 Total Family Income	-	−0.31*	0.00	0.05*	1.00				
6 Married Household	-	−0.19*	0.01	0.05*	0.51*	1.00			
7 Physical Activity	-	−0.03*	0.04*	0.04*	0.09*	0.07*	1.00		
8 Neighborhood Income / 50000	-	−0.37*	−0.02	0.06*	0.39*	0.23*	0.09*	1.00	
9 Body Mass Index (BMI)	-	0.05*	−0.09*	0.07*	−0.12*	−0.07*	0.00	−0.08*	1.00

* p < 0.05

**Table 3.** Linear regressions between neighborhood stress and body mass index in US youth

	Coefficient	Std. Err.	[95% Conf.	Interval]	*p*
**Model 1**					
Neighborhood Stress	0.620	0.040	0.542	0.697	< 0.001
**Model 2**					
Age	0.670	0.079	0.515	0.826	< 0.001
Male	−0.181	0.080	−0.338	−0.024	0.024
Total Family Income	−0.272	0.023	−0.316	−0.227	< 0.001
Married household	−0.488	0.108	−0.698	−0.277	< 0.001
Physical Activity	−0.042	0.018	−0.076	−0.007	0.017
Neighborhood Income / 50000	−0.472	0.107	−0.681	−0.263	< 0.001
Neighborhood Stress	0.078	0.047	−0.015	0.170	0.099
Black	1.010	0.113	0.789	1.232	< 0.001
**Model 3**					
Age	0.670	0.079	0.514	0.825	< 0.001
Male	−0.175	0.080	−0.332	−0.017	0.029
Total Family Income	−0.272	0.023	−0.316	−0.228	< 0.001
Married household	−0.492	0.108	−0.703	−0.281	< 0.001
Physical Activity	−0.041	0.018	−0.076	−0.007	0.019
Neighborhood Income / 50000	−0.481	0.107	−0.690	−0.272	< 0.001
Neighborhood Stress	0.145	0.056	0.035	0.255	0.010
Black	1.494	0.246	1.011	1.976	< 0.001
Neighborhood Stress x Black	−0.207	0.094	−0.390	−0.023	0.027
